# Analysis of fundamental frequency, jitter, shimmer and vocal intensity in children with phonological disorders

**DOI:** 10.1016/S1808-8694(15)31261-1

**Published:** 2015-10-20

**Authors:** Haydée F. Wertzner, Solange Schreiber, Luciana Amaro

**Affiliations:** 1 Full Professor, Course of Speech and Hearing Therapy, USP, Coordinator of the Speech and Hearing Laboratory of Investigation in Phonology.; 2 Speech and Hearing Therapist.; 3 Speech and Hearing Therapist. Master studies under course, School of Philosophy, Languages and Social Sciences, University of Sao Paulo.; Course of Speech and Hearing Therapy, Department of Physical Therapy, Speech and Hearing Therapy, Occupation Therapy, Medical School, University of Sao Paulo.

**Keywords:** language development disorders, speech acoustics, diagnosis

## Abstract

Phonological Disorder is a disturbance of primary manifestation of undefined causes that makes speech become unintelligible. The analysis of vocal parameters becomes important in the process of diagnosis of this disorder, since voice disorders could interfere in the production of speech sounds. **Aim:** The objective of this study was to verify vocal characteristics related to the intensity and fundamental frequency -F0- and their disturbance indexes - jitter and shimmer - in children with phonological disorders. **Study design:** clinical prospective with transversal cohort. **Material and Method:** There were 40 children, 20 of them with phonological disorders and 20 with no speech and language disturbances. Phonological exams with the ABFW infantile language test and spontaneous speech were applied. The Computer Speech Lab was used to record and perform acoustic analyses of the vowels /a/, /e/, /i/, through the vocal parameters: fundamental frequency, intensity, jitter and shimmer. **Results:** F0 - vowel /e/ was smaller, on average, in the Phonological Disorder Group and it was 126 Hz in the Control Group. To shimmer and jitter there was no evidence that the means of the Phonological Disorder Group were different from the ones of the Control Group (p= 0.191, p= 0.865, respectively). As for intensity, there was evidence that the average did not differ in the Phonological Disorder Group and the Control Group (p= 0.002). **Conclusion:** The frequency of the vowel /e/ was smaller in the Phonological Disorder Group. There was difference between the two groups regarding the means of intensity of vowels /a/, /e/ and /i/, smaller in the Phonological Disorder Group. No differences between the groups were found regarding the averages of jitter and shimmer.

## INTRODUCTION

Phonological disorders are affections to primary manifestation of undefined cause ^1^, which may turn the speech into intelligible production leading to misunderstood messages.

According to the survey performed among speakers of Brazilian Portuguese by Andrade et al. ^2^, about 54% of the children who were seen in the speech and hearing sector of Centro de Saude Escola Samuel Barnsley Pessoa had phonological and/or phonetic affections. Gierut ^3^ stated that the phonological disorders affect approximately 10% of the population of American children in pre-school and school age, and it is sufficiently severe in many cases to require clinical intervention in 80% of the cases.

The literature refers that to the proficiency of the phonological system by children involves the development of perception and production of a phonetic inventory, as well as phonological rules. Phonological rules correspond to the regularities that occur in the Phonology of a language ^4^.

Systematic simplifications of phonological rules that affect a class or sequence of sounds are named phonological processes. Phonological processes are expected within normal development, however as the child grows and develops, they no longer use them and acquire the rules similar to adults’ ^5^.

In children with typical development, phonological processes are naturally suppressed, whereas in children with phonological disorders, clinical intervention is required. To that end, a well-structured assessment with selection and administration of the right tests is mandatory ^6^.

Making use of such procedures, researchers intend to understand the characteristics of the phonological disorder, considering the unknown cause so as to classify possible subtypes. Following this line, Shriberg and Kwiatkowski 7, 8, 9 identified some etiological factors for the phonological disorder, which allows the division into four subtypes: unknown origin, otitis media, speech development apraxia (SDA), and psychosocial involvement.

In 1999, Shriberg^10^ proposed a new classification that suggested the existence of five subgroups with different etiologies: delay in speech of genetic origin, delay in speech with repetitive otitis media with effusion, speech delay with SDA, speech delay with implication of psychosocial development and, finally, type of residual mistakes with history of distortions.

### Factors related with the assessment for functional diagnosis

As phonological disorders are very common in the pre-school and school populations and they may be caused by different causes, investigations try to find specific descriptions for segmental and supra-segmental linguistic symptoms to relate them with specific characteristics of each cause correlated with the disorder. According to Lowe ^11^, segmental traits are referred to vowels and consonants that get together to form syllables, words and sentences, whereas supra-segmental or prosodic traits are elements that form the rhythm of a specific language or production.

To that end, in the speech assessment of phonological disorders, in addition to phonological analysis, we should include an analysis of cognitive, linguistic, neuromotor skills as well as the oral structure and functions, hearing, fluency, voice and supra-segmental aspects of speech 7, 12. We should highlight that the process of functional diagnosis explores the search for etiological factors and tries to separate the subtypes of phonological disorders, which is important for more precise clinical intervention.

### Aspects related with vocal quality

The segmental and supra-segmental aspects of vocal production are observed in the speech. The speech, considering that it is exclusive to a subject, becomes adjustable to the particularities of the speaker. Thus, each person may use variations of speed, height and intensity in their production. According to Peña-Brooks and Hedge^13^ the decrease and increase in height are essential to give melody to the sentence. Stress pattern, related with the combination of increased intensity, greater duration and higher frequency in syllables of a sentence emphasize some parts of the production, ensuring rhythm to the spoken language.

Abnormal prosodic characteristics have been described in the literature as a trait of speech development apraxia 9, 14, 15. As mentioned by Shriberg^10^, this condition is a subtype of phonological disorders. Thus, the assessment of prosody may contribute to the identification of this condition.

Shriberg et al. ^16^ presented a perceptual procedure (PVSP - Prosody-Voice Screening Profile) to assess the prosody and voice of spontaneous speech. These authors consider essential to assess vocal characteristics of children with phonological disorders, including aspects such as pitch, loudness and vocal quality.

The study of the speech prosodic aspects may be conducted by means of assessment of fundamental frequency (f0) variation, duration of syllables, words and other units and intensity ^17^.

According to Behlau and Pontes ^18^, vocal intensity is directly related with subglottic pressure of the air column. Subglottic pressure, in turn, depends on factors such as amplitude of vibration and tension of vocal folds, more specifically the glottic resistance.

Variations of intensity, however, also depend on frequency ^19^. To Behlau and Pontes ^18^, high voices tend to be more intense, because the increase in laryngeal tonus generates higher glottic resistance and, consequently, more intensity.

The analysis of vocal parameters become important in the process of diagnosis of phonological disorders, given that vocal disorders and articulation normally coexist ^20^, given that the former may lead to difficulties of sound in the latter.

The voice may be assessed subjectively (perceptual-auditory assessment) and/or objectively to help with acoustic analysis equipment. According to Gurgueira ^21^ the acoustic analysis enables determination of number, frequency and amplitude (intensity) of vibrations to form a complex sound.

The most important vocal acoustic parameters for clinical use are measurements of noise, vocal extension profile, acoustic spectrography, fundamental frequency and perturbation index - jitter and shimmer ^22^.

According to Behlau et al. ^22^ fundamental frequency is determined physiologically by the number of cycles that the vocal folds make in a second, and they are the natural result of the length of these structures.

Jitter and shimmer represent the variations that occur in the fundamental frequency. Whereas jitter indicates the variability or perturbation of fundamental frequency, shimmer refers to the same perturbation, but it is related to amplitude of sound wave, or intensity of vocal emission. Jitter is affected mainly because of lack of control of vocal fold vibration and shimmer with reduction of glottic resistance and mass lesions in the vocal folds, which are related with presence of noise at emission and breathiness ^22^.

Some studies were developed to compare perceptually and acoustically the voice of children with and without communication affections, such as the one performed by Shriberg and Kwiatkowski ^9^ who used the perceptual procedure PVSP - Prosody-Voice Screening Profile to compare a group of 64 children aged 3 to 6 years, with phonological disorders, whose speech mistakes were severe enough to interfere in the intelligibility and a group of 71 children aged 3 to 5 years with normal speech development, studied by Miller ^27^. Both groups presented similar results in speech rate, pitch and resonance. However, 17.8% of the children with phonological disorders versus 1.4% of the normal children were classified as having perceptual and discussable involvement of stress pattern; 30.7% versus 2.8% in loudness, and 48.8% vs. 23.8% in laryngeal vocal quality aspects.

There are few studies about supra-segmental acquisition and relations between vocal parameters and phonological disorders in children. Studies in this area are extremely important to enable advances in search for etiology and ensure more effective intervention and early identification of the disorder.

Thus, the present study intended to study the vocal characteristics related to intensity and fundamental frequency and their perturbations indexes - jitter and shimmer, in children with phonological disorders.

## MATERIAL AND METHOD

The present study was approved by the Ethics and Research Committee HC-FMUSP (nº 00/09220-3) and sponsored by FAPESP (process nº 02/03102-4). The people responsible for the children signed the Informed Consent Term.

The study comprised 40 children aged 4 to 10.2 years, both genders, residents in Sao Paulo.

The group with phonological disorder (GTF) comprised 29 subjects, 9 female and 11 male subjects, seen in the Laboratory of Phonology (LIF), Department of Physical Therapy, Speech and Hearing Therapy, Occupation Therapy, Medical School, University of Sao Paulo. They were selected after the diagnostic process that included language tests ABFW28, spontaneous speech test, oral myofunctional system analysis, phonological awareness and audiological assessment.

The control group (GC) comprised 20 subjects without speech or language affections, from schools in the region of Butanta, Sao Paulo, 9 female and 11 male subjects. To select then, we used a questionnaire that the parents answered to check whether they had any complaint concerning speech or language. Next, we applied the phonology test of language ABFW29 and the spontaneous speech test. Thus, we included in the GC only subjects who presented phonological development within the expected level for the age without complaints related to language development.

All tests were recorded in digital audio tape (DAT Foster D-S Digital Master Record) and recorded in digital video (Sony CCD-TRV66).

To acoustically analyze the studied parameters - fundamental frequency (F0), jitter, shimmer and intensity - we used the Computer Speech Lab (CSL), manufactured by Kay Elemetrics - model 4300B, and unidirectional microphone brand Shure, model SM-58.

### Procedures

The analysis of F0 (Hz), jitter (%), shimmer (dB) and intensity (dB HL) was performed with isolated production and sustained vowels /a/, /e/, and /i/.

Subjects remained seated and were instructed to produce vowels at comfortable intensity and height, with the microphone at 10cm from their mouth. Each vowel was produced 3 times, and they were directly recorded by the Computerized Speech Lab (CSL).

To neutralize the effects of vocal attach, the beginning of the recording of each vowel was discarded. Samples were edited to present the same duration in all subjects, or 2 seconds.

Each one of the three sustained vowel productions were individually analyzed for each one of the studied parameters. The final value of the measures of each of the vowels was the mean obtained from the analysis of each one of the vowels separately.

After data collection, we adjusted in the computer the command of the sampling rate for 10,000 Hz.

To reach values of F0, jitter and shimmer, vowel samples were made through commands of CSL. The calculation of intensity was also made in CSL, which provided intensity in three moments - beginning of emission (0 second), half emission (1 second) and end of emission (2 seconds). The final value of the obtained intensity was the mean of initial, mid and final values of each vowel.

## RESULTS

The descriptive analysis demonstrated that F0 of vowels /a/ and /i/ were on average similar in both groups, whereas F0 of vowel /e/ was smaller on average for GTF (237 Hz for GC and 126 Hz for GTF). Jitter in all vowels presented similar distribution for both groups. As to shimmer, only in vowel /e/ it was higher than the median in GTF (0.339 dB for GC and 0.486 dB for GTF).

As to intensity, we observed that vowels /a/, /e/ and /i/ presented smaller values than the median for GTF. Results may be better visualized in Tables 1 and 2.

**Table 1:** Descriptive analysis - GC

Variables	N	Mean	Median	Standard deviation	Minimum	Maximum	Q1	Q3
Fo /a/ (Hz)	20	243	240	24	206	281	225	264
Fo /e/ (Hz)	20	237	241	31	148	285	218	262
Fo /i/ (Hz)	20	229	245	46	153	281	171	265
Jitter /a/ (%)	20	1,551	1,338	0,906	0,909	5,158	1,043	1,653
Jitter /e/ (%)	20	1,678	1,331	1,303	0,814	6,326	1,078	1,547
Jitter /i/ (%)	20	1,113	1,008	0,368	0,642	2,239	0,868	1,313
Shimmer /a/ (dB)	20	0,610	0,541	0,187	0,393	1,013	0,445	0,743
Shimmer /e/ (dB)	20	0,375	0,339	0,170	0,185	0,918	0,262	0,461
Shimmer /i/ (dB)	20	0,465	0,426	0,243	0,129	1,298	0,363	0,524
Intensity - /a/ (dB)	20	73,8	73,8	2,2	69,7	78,9	72,0	75,3
Intensity - /e/ (dB)	20	75,8	75,7	2,2	70,8	79,4	74,4	77,5
Intensity - /i/ (dB)	20	75,5	75,3	2,4	71,5	81,5	74,1	77,1

Key:

GC - control group

F0 - fundamental frequency

N - total number of subjects

Q1 - first quartile

Q3 - third quartile

**Table 2:** Descriptive analysis - GTF

Variables	N	Mean	Median	Standard deviation	Minimum	Maximum	Q1	Q3
Fo /a/ (Hz)	20	246	246	23	193	284	232	262
Fo /e/ (Hz)	20	126	126	12	100	146	118	136
Fo /i/ (Hz)	20	228	237	42	171	285	183	265
Jitter /a/ (%)	20	1,609	1,247	0,899	0,810	3,825	0,986	2,063
Jitter /e/ (%)	20	1,965	1,395	1,914	0,673	8,732	0,985	1,722
Jitter /i/ (%)	20	1,331	1,107	0,816	0,706	4,243	0,834	1,370
Shimmer /a/ (dB)	20	0,610	0,576	0,251	0,282	1,369	0,420	0,735
Shimmer /e/ (dB)	20	0,501	0,486	0,235	0,195	1,123	0,303	0,653
Shimmer /i/ (dB)	20	0,526	0,486	0,216	0,150	0,864	0,337	0,709
Intensity - /a/ (dB)	20	72,2	72,2	2,02	69,1	76,2	70,5	73,8
Intensity - /e/ (dB)	20	73,5	74,5	2,9	68,1	80,2	71,0	75,1
Intensity - /i/ (dB)	20	72,7	72,6	3,3	66,1	78,0	70,3	75,1

Key:

GC - control group

F0 - fundamental frequency

N - total number of subjects

Q1 - first quartile

Q3 - third quartile

**Figure 1:**
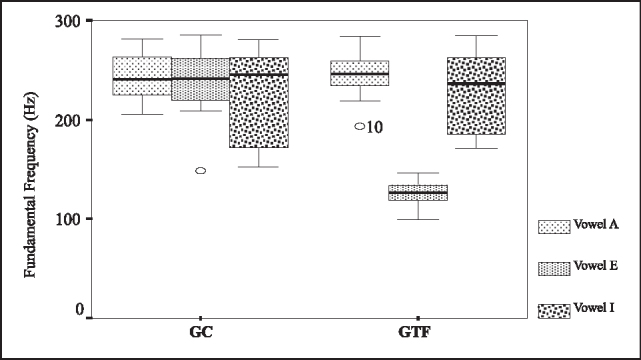
Box plots of F0 for vowels /a/, /e/ and /i/ in GC and GTF. Key: GC- control group GTF - group with phonological disorder

To perform the inference analysis of F0 we used the non-parametric Mann-Whitney test. Table 3 shows a level of significant of 5%, concluding that only fundamental frequency of vowel /e/ was different between GTF and GC. Figure 1 is a box plot related to the comparison between GC and GTF and variable F0.

**Table 3:** Comparison between GC and GTF - fundamental frequency.

Vowel	Descriptive level
A	0,637
E	< 0,0001
I	0,758

Key:

GC- control group

GTF - group with phonological disorder

**Figure 2:**
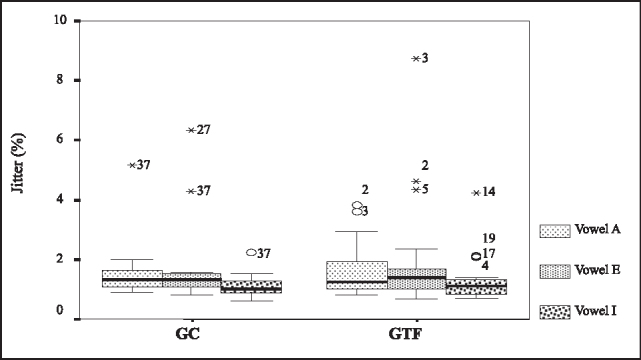
Box plots of jitter for vowels /a/, /e/ and /i/ in GC and GTF.

**Figure 3:**
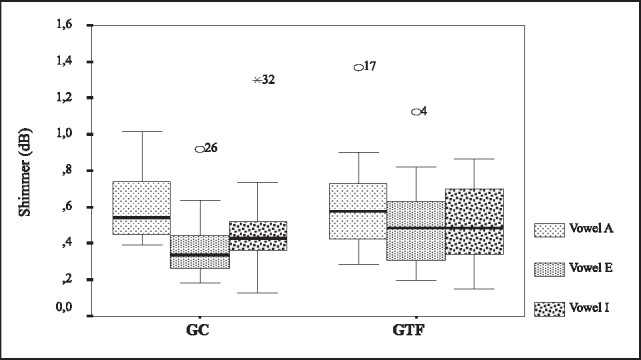
Box plots of shimmer for vowels /a/, /e/ and /i/ in GC and GTF. Key: GC- control group GTF - group with phonological disorder

To compare the means of shimmer and jitter we used the Analysis of Variance with two fixed factors and repetitive measures in a factor after the transformation of the variable response according to the box plot. To shimmer and the level of significance of 5%, we concluded that there was no evidence that the means of GTF were different from GC (p=0.191). For jitter, the test did not show differences between GC and GTF (p=0.865). Figures 2 and 3 refer to Box plots that compared GC and GTF concerning the variables of shimmer and jitter.

**Figure 4:**
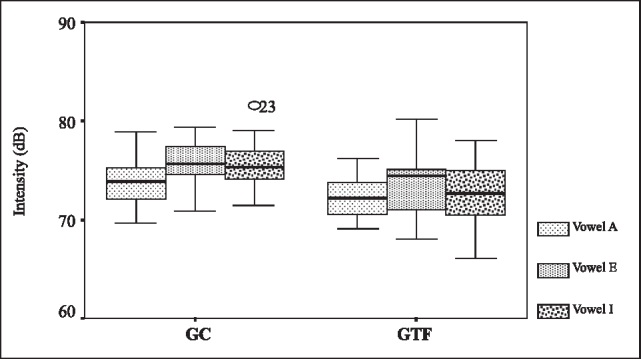
Box plots for intensity of vowels /a/, /e/ and /i/ in GC and GTF.

Finally, for intensity, we used the Analysis of Variance with two fixed factors and repetitive measures of one factor. At a level of significance of 5%, we detected evidence that the mean of intensity would differentiate GTF from GC (p=0.002). Figure 4 shows box plots comparing GC and GTF in relation to intensity.

## DISCUSSION

The results found in this study showed that both children in the GC and in the GTF presented values of F0 similar to other studies. Thus, mean values of F0 for vowel /a/ were between 243 and 246 Hz and for vowel /i/ between 229 and 228 Hz, values close to those found by Hasek et al.^23^ , in which mean fundamental frequency values found for male subjects aged 5 years were 247.5 Hz, 6 years 262.5 Hz, 7 years 234.2 Hz, 8 years 235.6 Hz, 9 years 230.4 Hz and 10 years 228.9 Hz. For female subjects, the following values were found for 5 years 257.7 Hz, 6 years 254.3 Hz, 7 years 261.7 Hz, 8 years 264 Hz, 9 years 246.7 Hz and 10 years 253.7 Hz.

The results found by Navas ^24^ indicated mean values between 298.1 and 290.9 Hz for male and 299.8 and 290.9 Hz for female. Behlau^26^ observed that the value of F0 for children aged 8 to 11 years was 236 Hz and Awan and Mueller ^25^ detected in spontaneous speech 243 Hz for female subjects and 240 Hz for male subjects.

Only vowel /e/ differentiated the two groups (in GC the mean value found was 237 Hz, whereas in the GTF this value was 126Hz), a fact that may be related with the configuration of the vocal tract required for sound production. The differences in vocal tract may be resulting from the adapted movements children who have phonological disorders make to execute their productions. It is interesting to emphasize that this finding should be properly investigated to check the possibility of being used as a differential element in children with this disorder.

Even though there were no significant differences between GC and GTF for jitter and shimmer, the values found were below those reported in another study with Brazilian children, by Behlau ^26^, in which jitter was 2.3% and shimmer was 2.5 dB.

This fact could have been influenced by the use of different equipment for the acoustic analysis.

As to intensity, we observed statistically significant differences between the two groups, in which GTF presented mean intensities lower than those in the GC. This finding may be related with psychosocial aspects that interfered in the communication of children with phonological disorder.

It is important to emphasize that it was difficult to compare our acoustic findings with the findings of other studies, given that these studies focused on adults and children with affections and different communication disorders and not phonological disorders. Thus, the variation of methodologies and equipment for the acoustic analysis using these studies limits the possible comparison between studies.

However, the results found in the present study point to the fact that these children with phonological disorders compared to children without disorders do not present any abnormality that affects the vocal folds, either muscle or neural activity involved with phonation, either lesions that may cause increase in aperiodicity of vocal fold vibration, which reflect the increased values of jitter. As to shimmer, the study indicated that the characteristics such as reduction of glottic resistance, vocal fold mass lesions and greater noise at production, factors that could lead to affections of shimmer values, were not necessarily found.

## CONCLUSION

The study showed that, in the comparison between GC and GTF, F0 of vowel /e/ is lower than for other vowels in GTF, whereas in GC all vowels had similar F0 values. Another detected difference between the groups was the mean intensity of vowel /a/, /e/ and /i/, which were lower in GTF.

We did not find statistically significant differences between the groups concerning means of jitter and shimmer of vowels. For the vowel effect, we observed that the mean of jitter of vowel /i/ was lower than for vowels /a/ and /e/ and the mean of shimmer of the vowel /a/ was higher than for vowels /e/ and /i/.

Similarly, F0 of vowel /e/ and intensity were acoustic measurements that led to differentiation between the groups, which should be further investigated. As pointed out by the studies by Shriberg 8, 10, 14, these characteristics may be used to complement the diagnosis of phonological disorders and have to be further studied.
